# Erratum

**DOI:** 10.1080/14686996.2016.1176342

**Published:** 2016-04-19

Tsao C-W and Lee Y-P. Magnetic microparticle-polydimethylsiloxane composite for reversible microchannel bonding. *Science and Technology of Advanced Materials* 2016;17:2-11. 10.1080/14686996.2016.1140301

Lei Y, Ma W, Sun L, Wu J, Dai Y and Morita K. Removal of B from Si by Hf addition during Al–Si solvent refining process. *Science and Technology of Advanced Materials* 2016;17:12-19. 10.1080/14686996.2016.1140303

Scopece D, Döbeli M, Passerone D, Maeder X, Neels A, Widrig B, Dommann A, Müller A and Ramm J. Silicon etch with chromium ions generated by a filtered or non-filtered cathodic arc discharge. *Science and Technology of Advanced Materials* 2016;17:20-28. 10.1080/14686996.2016.1140308

Gutierrez-Urrutia I, Archie F, Raabe D, Yan F-K, Tao N-R and Lu K. Plastic accommodation at homophase interfaces between nanotwinned and recrystallized grains in an austenitic duplex-microstructured steel. *Science and Technology of Advanced Materials* 2016;17:29-36. 10.1080/14686996.2016.1140302

Kang J, Kim H, Saito N and Lee M-H. A simple synthesis method for nanostructured Co-WC/carbon composites with enhanced oxygen reduction reaction activity. *Science and Technology of Advanced Materials* 2016;17:37-44. 10.1080/14686996.2016.1140305

Boota M, Houwman EP, Dekkers M, Nguyen MD, Vergeer KH, Lanzara G, Koster G and Rijnders G. Properties of epitaxial, (001)- and (110)-oriented (PbMg_1/3_Nb_2/3_O3)_2/3_-(PbTiO_3_)_1/3_ films on silicon described by polarization rotation. *Science and Technology of Advanced Materials* 2016;17:45-57. 10.1080/14686996.2016.1140306

Sun L, Wang H, Eid K and Wang L. Shape-controlled synthesis of porous AuPt nanoparticles and their superior electrocatalytic activity for oxygen reduction reaction. *Science and Technology of Advanced Materials* 2016;17:58-62. 10.1080/14686996.2016.1140307

Lutz T, Veissier L, Thiel CW, Woodburn PJT, Cone RL, Barclay PE and Tittel W. Effects of fabricationmethods on spin relaxation and crystallite quality in Tm-doped Y_3_Al_5_O_12_ powders studied using spectral hole burning. *Science and Technology of Advanced Materials* 2016;17:63-70. 10.1080/14686996.2016.1148528

Wang D, Guan K, Bai Z and Liu F. Facile preparation of acid-resistant magnetite particles for removal of Sb(III) from strong acidic solution. *Science and Technology of Advanced Materials* 2016;17:80-88. 10.1080/14686996.2016.1145530

When the above articles were first published online, the graphical abstracts were missing. These have now been added to the online versions.

Taylor & Francis apologises for these omissions.

